# Reasons for prolonged time for diagnostic workup for stage I-II lung cancer and estimated effect of applying an optimized pathway for diagnostic procedures

**DOI:** 10.1186/s12913-019-4517-z

**Published:** 2019-09-18

**Authors:** Trine Stokstad, Sveinung Sørhaug, Tore Amundsen, Bjørn H. Grønberg

**Affiliations:** 1 Faculty of Medicine and Health Sciences, Department of Clinical and Molecular Medicine, NTNU, Norwegian University of Science and Technology, PO Box 8905, MTFS, NO-7491 Trondheim, Norway; 2 Department of Gynecology, St. Olavs hospital, Trondheim University Hospital, PO Box 3250, Sluppen, NO-7006 Trondheim, Norway; 3 Faculty of Medicine and Health Sciences, Department of Circulation and Medical Imaging, NTNU, Norwegian University of Science and Technology, PO Box 8905, MTFS, NO-7491 Trondheim, Norway; 4 Department of Thoracic Medicine, St. Olavs hospital, Trondheim University Hospital, PO Box 3250, Sluppen, NO-7006 Trondheim, Norway; 5 Cancer Clinic, St. Olavs hospital, Trondheim University Hospital, PO Box 3250, Sluppen, NO-7006 Trondheim, Norway

**Keywords:** Pathway, Timeliness, Diagnostic efficacy, Organization

## Abstract

**Background:**

Minimizing the time until start of cancer treatment is a political goal. In Norway, the target time for lung cancer is 42 days. The aim of this study was to identify reasons for delays and estimate the effect on the timelines when applying an optimal diagnostic pathway.

**Methods:**

Retrospective review of medical records of lung cancer patients, with stage I-II at baseline CT, receiving curative treatment (*n* = 100) at a regional cancer center in Norway.

**Results:**

Only 40% started treatment within 42 days. The most important delays were late referral to PET CT (*n* = 27) and exercise test (*n* = 16); repeated diagnostic procedures because bronchoscopy failed (*n* = 15); and need for further investigations after PET CT (*n* = 11). The time from referral to PET CT until the final report was 20.5 days in median. Applying current waiting time for PET CT (≤7 days), 48% would have started treatment within 42 days (*p* = 0.254). “Optimal pathway” was defined as 1) referral to PET CT and exercise test immediately after the CT scan and hospital visit, 2) tumor board discussion to decide diagnostic strategy and treatment, 3) referral to surgery or curative radiotherapy, 4) tissue sampling while waiting to start treatment. Applying the optimal pathway, current waiting time for PET CT and observed waiting times for the other procedures, 80% of patients could have started treatment within 42 days (*p* < 0.001), and the number of tissue sampling procedures could have been reduced from 112 to 92 (− 16%).

**Conclusion:**

Changing the sequence of investigations would significantly reduce the time until start of treatment in curative lung cancer patients at our hospital and reduce the resources needed.

## Background

Long intervals for completion of diagnostic workup and start of treatment causes distress among cancer patients [1], and is conceived as a medical risk that may negatively impact survival [2–4]. Thus, it is a political goal that diagnostic workup for suspected cancer should be performed efficiently and with no delays except those who are due to medical reasons. Consequently, programs for timely care have been developed, e.g. the two-week-wait referral pathway in the UK [5, 6], and the national cancer pathway intervention in Denmark [7, 8].

Diagnostic workup for lung cancer has become complex due to the increasing number of treatment options. More patients are eligible for potentially curative therapy due to less invasive surgery and advanced radiotherapy techniques but require more extensive tissue sampling for correct staging of disease, and targeted therapies are selected according to molecular profiling of tissue samples. Thus, a multidisciplinary approach including sufficient resources for imaging, tissue sampling and analyses is required, necessitating a good organization across departments and health care levels.

In Norway, current national guidelines recommend that a patients’ first hospital visit should take place within 7 calendar days after the hospital receives a referral letter for suspected lung cancer; a treatment decision should be made within 28 days; systemic therapy should start within 35 days; and surgery or radiotherapy within 42 days [9]. It is a goal that 70% start treatment within these target timeframes. The timeframes are consensus-based and were met with skepticism. A common opinion among physicians was that it would not be possible to achieve these goals with allocating more resources to the hospitals.

There are few studies of the logistics of lung cancer diagnostic work-up, and mechanisms for delays are poorly described [10]. In a previous study, we found that only a minority of lung cancer patients started treatment within the recommended timeframes at our hospital [11]. There was a need to better understand why delays occurred, and to assess whether the recommended timeframes could realistically be met without increasing the budgets of the involved hospital departments. The aims of this study were to identify reasons for delays, define an optimal pathway for diagnostic procedures, and estimate the effect on the timelines of applying this pathway. Due to the heterogeneity and complexity of lung cancer patients in general, we limited the present study to patients presenting with stage I-II on the base-line CT scan who were eligible for potentially curative treatment.

## Methods

### Study setting

St. Olav’s Hospital, Trondheim University Hospital, in Trondheim, Norway, is the primary hospital for 380,000 people, the regional cancer center for the Central Norway Health Region with a population of approximately 700,000 people, and the only hospital in the region to offer lung cancer surgery and positron emission tomography computer tomography (PET CT) (since October 2013). Lung cancer diagnosis take place at the Department of Thoracic Medicine by pulmonologists specializing in lung cancer diagnosis and treatment, and they also offer systemic therapy; the Cancer Department provides radiotherapy, oncologists are trained in both medical oncology and radiotherapy; and surgery take place in the Department of Cardio-Thoracic Surgery. A tumor board of pulmonologists, thoracic surgeons, oncologists, thoracic radiologists, specialists in nuclear medicine, pathologists and a patient coordinator, meets every week.

### Study design, patients and data collection

We performed a retrospective analysis of the individual hospital medical records of all lung cancer patients presenting with stage I-II disease at the baseline CT scan, who were diagnosed at the Department of Thoracic Medicine, and who underwent surgery or curative radiotherapy at St. Olavs hospital between January 1, 2011 until December 31, 2013. More details about the conduct of our study is included in a previous publication [11]. Patients with a delay of ≥3 days caused by comorbidity, intercurrent disease, or the patients’ wish were excluded. Stage of disease was assessed according to the 7th edition of the TNM classification of lung cancer [12].

Exercise tests included stair-climbing test, six-minute walk-test, and cardiopulmonary exercise test [13]. We defined tissue diagnostic method as either bronchoscopy, bronchoscopy and endobronchial ultrasound transbronchial aspiration (EBUS-TBNA), or transthoracic needle biopsy (TTNB); and that the method “failed” when the diagnosis was confirmed by subsequently using another method, or the patient underwent both bronchoscopy and bronchoscopy with EBUS-TBNA.

We registered a) the date when a referral letter for suspected lung cancer was registered at the Department of Thoracic Medicine, or the date when diagnostic workup for suspected LC was initiated in a patient with a single pulmonary nodule who had been previously observed (“Receiving a referral letter”); b) the date of the first appointment with a pulmonologist (“First consultation”); c) for each diagnostic work-up procedure: c) type of procedure, d) date of referral to the procedure, e) the date it took place, and f) the date when the result of a procedure was documented in the patient’s medical record; g) the date a treatment decision was documented in the patient’s medical record (“Treatment decision”); h) the date of surgery or first day of radiotherapy or chemotherapy (“Start of treatment”). “Time to treatment” was defined as interval in calendar days from receiving a referral letter for suspected lung cancer until start of treatment; “Timely”, ≤ 42 days; “Untimely”, > 42 days. Finally, we calculated the median times for referral to each diagnostic procedure until the final report was available.

### Data analysis

The sequences of actions were ordered and intervals in calendar days were calculated: from receiving a referral letter until first consultation; from first consultation until referral for diagnostic work-up procedure(s); from referral to a procedure until the result was available in the electronic medical record; from the result of a procedure until this result was actioned upon (by referral to another procedure or making a treatment decision); from treatment decision until start of treatment.

Models for simulating improvements were built by changing the sequence of actions. The numbers who could start timely treatment were compared using logistic regression. Analyses were performed using the Stata/IC 14.2 package for Windows.

## Results

### Patient characteristics

**Fig. 1:**
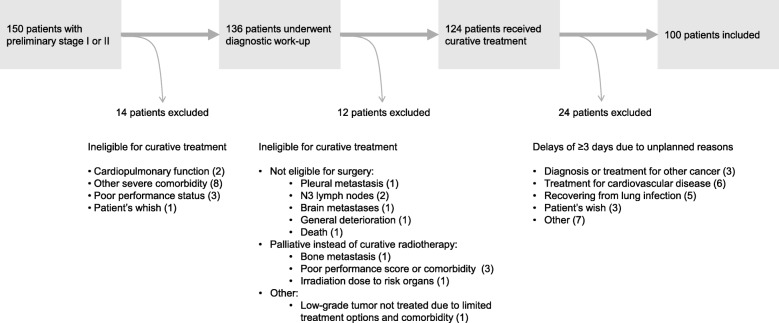
Patient selection. Preliminary stage was defined as TNM stage according to the baseline CT scan

Four hundred fifty-four patients were diagnosed with lung cancer between January 1, 2011 and December 31, 2013. Five patients declined inclusion [11], and among the other 449 patients, 150 presented with preliminary stage I or II. Twenty-six patients were excluded since they were ineligible for curative treatment; and another 24 were excluded because they experienced delays of ≥3 days due to medical reasons or patient’s whish (Fig. 1). Thus, 100 patients were included in the present analyses. Median age was 70 (54–84), 77% had NSCLC, and 63% were women (Table 1).

**Table 1:** Baseline characteristics

	Included *N* = 100		Unplanned delay *n* = 24		Ineligible of curative treatment *n* = 26	
Median age (range)	70	(54–84)	70.5	(56–86)	81	(58–89)
Age ≥ 75 years	32	(32%)	9	(38%)	19	(73%)
Women	63	(63%)	12	(50%)	13	(50%)
Stage I	72	(72%)	16	(67%)	10	(38%)
Stage II	20	(20%)	7	(29%)	8	(31%)
Stage III	8	(8%)	1	(4%)	3	(12%)
Stage IV					5	(19%)
Surgery	76	(76%)	15	(63%)		
Curative radiotherapy^a^	8	(8%)	1	(4%)		
Stereotactic radiotherapy^b^	16	(16%)	8	(33%)		
Palliative treatment					12	(46%)
NSCLC	77	(77%)	18	(75%)	11	(42%)
SCLC	6	(6%)	1	(4%)	2	(8%)
Another primary lung cancer	5	(5%)	1	(4%)	1	(4%)
No tissue diagnosis	12	(12%)	4	(17%)	12	(46%)

^a^Includes chemo-radiotherapy in limited disease SCLC

^b^In T1-2 N0 NSCLC

### The observed pathway

Most patients had a CT of the chest and upper abdomen before referral. Referrals were reviewed by an experienced pulmonologist and the first hospital visit including a spirometry and a bronchoscopy was scheduled. Images, pathology results and physical tests were presented at the weekly tumor board meeting during which the need for supplementary diagnostic procedures and treatment decisions were made.

### Causes for delayed treatment

**Fig. 2:**
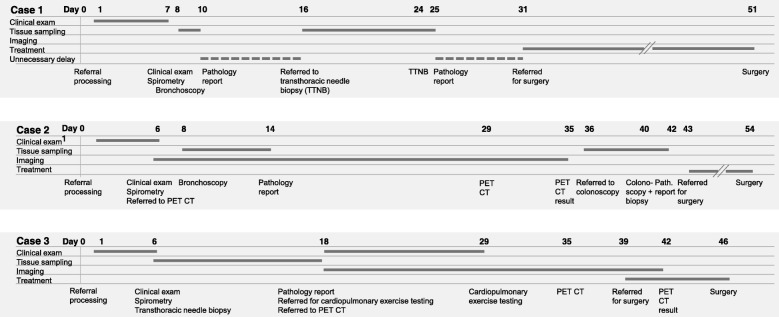
Patient cases demonstrating some common reasons why treatment was delayed: several procedures were performed when it was evident that the last procedure was most likely to succeed (Case 1); a late PET CT revealed lesions that caused sequential diagnostic procedures (Case 2); unnecessary delays because the pathology reports were not acted upon (and the patients were not informed) until several days after they were completed (Case 1, marked with a stapled line); late referral to PET CT and cardiopulmonary exercise testing (Case 3); long waiting time for pathology report (Case 2 and 3), PET CT (Case 2 and 3), and cardiopulmonary exercise testing (Case 3); and long waiting time for treatment (Case 1)

We found several factors that led to delayed start of treatment. We have presented the actual pathway for three patients in Fig. 2 to illustrate some of the most common causes for delay. The most important causes for delay were:

1. Disease stage was not correctly assessed before the first hospital appointment took place because the pulmonologist who reviewed the referral letter did not assess that the quality of the initial CT scan was too poor, too old, or missed the upper abdomen (*n* = 8, caused a delay of median 15.5 days, range: 2–98 days). This was usually pointed out by the chest radiologist, who often did not see the images until the tumor board meeting. Consequently, the radiology report was not completed when the first hospital visit took place (*n* = 39, caused a delay of median 2 days, range: 1–55 days).
2. Patients were not referred to a PET CT at the first consultation (*n* = 27, median of 8 days later, range: 1–36 days), partly because the radiology report of the CT scan was not available.
3. Patients were not referred for an exercise test at the first hospital visit (*n* = 16, median of 10 days later, range: 2–28 days). In total, 21 patients underwent exercise testing.
4. Patients underwent subsequent tissue procedures because an attempt of sampling tumor through bronchoscopy failed when a transthoracic CT guided biopsy was the method that produced a diagnosis (*n* = 15). The method for obtaining a diagnostic tissue sample was bronchoscopy in 27 cases (at the second attempt in three cases) and a transthoracic needle biopsy in 19 (following a negative bronchoscopy in 15 cases). Fifty-four patients were referred to treatment without a definitive cancer diagnosis (42 to surgery, 11 to stereotactic radiotherapy and one to conventional radiotherapy). Of these, 22 underwent no tissue sampling procedures, 27 underwent one negative bronchoscopy, and two attempts were made in five cases.
5. Need for additional diagnostic procedures due to findings on PET CT (*n* = 12; FDG upload in mediastinal lymph nodes (*n* = 3), the thyroid gland (*n* = 2), parotid gland (*n* = 1), pharynx (*n* = 1), small intestine (*n* = 1), colon (*n* = 2), heart (*n* = 1), and genitals (*n* = 1)).
6. Incomplete investigation before the patient was discussed at the tumor board (10 patients were referred to PET CT, and 6 to exercise testing). Thus, the treatment decision was delayed (*n* = 16).
7. Interval from the hospital received a referral letter until the first hospital appointment exceeded 7 days for unexplained reasons (*n* = 50). Of these, 18 patients waited 14 days or more.
8. Long waiting time for PET CT. When the study was conducted, patients had to be referred to other hospitals for PET CT, and the median time until the PET CT reports were available was 20.5 days (range: 7–49). A PET CT scanner was installed in our hospital in October 2013, and the current waiting time is now 7 days or less.
9. Long waiting time from a tissue sampling procedure took place until the pathology report was completed (median of 4.5 days, range: 0–14 days). Furthermore, patients were routinely given an appointment for information about the pathology report 1–2 weeks after the tissue sampling procedure, which caused further delays when the sampling was unsuccessful.
10. Other important delays occurred due to waiting time for cardiopulmonary exercise testing (*n* = 9, median of 11 days, range: 1–19 days); and waiting time for a repeated tissue sampling procedure (median of 8 days, range: 1–14 days).
11. The median interval from referral to treatment until surgery was 13 days (range: 4–48 days), and until radiotherapy 22.5 days (range: 6–37 days).

We also found that two patients were medically operable but received chemo-radiotherapy due to SCLC. In all other patients, there was a medical reason when radiotherapy was chosen instead of surgery.

### Time to treatment

**Fig. 3:**
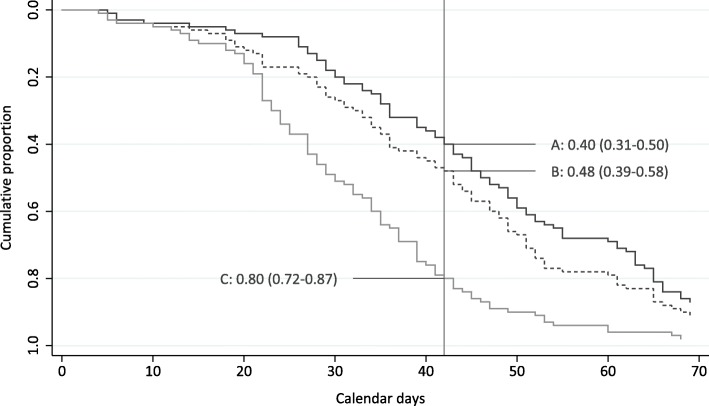
Time to treatment in calendar days from receiving a referral letter for suspected lung cancer in lung cancer patients with stage I-II on the baseline CT scan and who received curative treatment. A) observed timelines; B) estimated timelines when applying current waiting time for PET CT (≤ 7 days); C) estimated timelines when applying the optimal sequence of procedures and current waiting times for PET CT. The reference line at 42 days refer to the Norwegian Guidelines for timely lung cancer treatment

The median time to treatment was 46.5 days (5–145), and 40% (95% CI: 31 to 50%) of patients started treatment within the recommended 42 days (Fig. 3).

When applying the current waiting time for PET CT (≤7 days), the proportion of patients who could have started timely treatment increased to 48% (95% CI: 39 to 58%) (*p* = 0.255) (Fig. 3).

Based on our analyses, we defined the following optimal pathway:

1. If a CT of the chest and upper abdomen is not performed before, it should take zero days from a referral letter is received until referral for a CT scan.
2. In patients who are considered fit for curative treatment, it should take zero days from the first consultation until referral to PET CT.
3. In patients with reduced pulmonary function it should take zero days from the first consultation until referral for exercise testing.
4. Patients should be discussed at a tumor board meeting immediately after completion of exercise tests and PET CT to a) decide how tissue sampling for both diagnostic and staging purposes should be performed; b) make a treatment decision.
5. It should take zero days from the tumor board meeting until referral to a procedure using a method that is suitable for simultaneous diagnosis and staging.
6. It should take zero days from the tumor board meeting until referral to treatment.

**Fig. 4:**
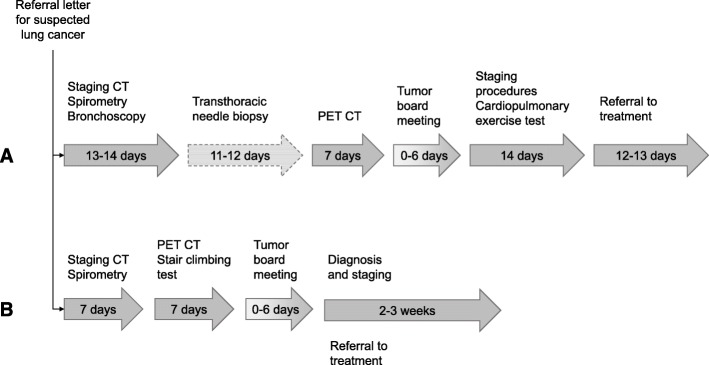
**a** Observed pathway. The optimal method for obtaining a tissue diagnosis was not always performed first, which led to more procedures. The most important reason for delays was that patients were referred for a PET CT too late during the diagnostic workup. The other main reason for delay was that exercise tests were not performed until after the tumor board discussions. Consequently, a treatment decision could often not be made during the tumor board meeting. **b** Our suggestions for an optimal pathway including median times for each procedure observed in our study cohort. The main points are that a PET CT and exercise test should be performed before the tumor board meeting and tissue sampling, and that patients should be referred for the most likely treatment before the results of the final diagnostic procedures are completed since the treatment seldom changes after the PET CT (in 2% of cases in our cohort)

Applying this optimal pathway, current waiting time for PET CT (≤7 days) and observed timelines for the other procedures, the proportion of patients who could start treatment within 42 days would increase to 80% (95% CI: 72 to 87%) (*p* < 0.001) (Fig. 3), and the number of tissue sampling procedures would have been reduced with 16% (from 112 to 92 procedures). Applying the optimal pathway, current waiting time for PET CT and median observed intervals for each diagnostic procedure, 93% patients could have started treatment within 42 days (Fig. 4).

## Discussion

We have previously found that few lung cancer patients started treatment within the recommended national timeframes at our hospital [11]. In the present study, we analyzed the individual patients’ pathways in order to investigate the reasons for delay, including patients who presented with stage I-II disease on the baseline CT scan who received curative treatment. We found that only 40% of patients started treatment within the recommended timeframe in Norway of 42 days, and suboptimal planning was an important explanation for this. Specifically, the main reasons were that patients were referred to a PET CT late during the diagnostic workup, leading to unnecessary delay and sequential procedures; the optimal method for tissue sampling was not chosen first, leading to repetition of procedures; and exercise tests were not performed before the patient was discussed at the tumor board meeting, leading to delayed treatment decision. There were long waiting times for PET CT during the study period, but when applying current waiting time for PET CT (≤7 days), the proportion of patients who could start treatment within 42 days only increased to 48%. Based on our analyses, we defined a more optimal sequence of actions. By applying this optimal pathway and current waiting time for PET CT, the proportion of patients who would have started treatment within 42 days increased to 80% and the number of diagnostic procedures would have decreased with 16%. Thus, implementing a more optimal pathway could improve timeliness of treatment and save resources.

Several interventions aiming at improving the timeliness of diagnostic workup and start of treatment have been proposed - including care pathways; patient navigators; fast-track programs; and different multidisciplinary decision making procedures [14]. Some studies indicate that such interventions may lead to improvement, but most include allocation of more resources and the exact mechanisms leading to improvement are poorly described [15–24]. Some have investigated the impact of different medical approaches. In a randomized trial of patients with suspected stage I-IIIA lung cancer, performing endobronchial ultrasound transbronchial needle aspiration (EBUS-TBNA) as the first procedure led to fewer procedures and shorter time until a treatment decision was made when compared to bronchoscopy or a transbronchial needle biopsy as the first procedure [25]. Similarly, a prospective study of tumors that based on chest CT were accessible to EBUS-TBNA showed that EBUS-TBNA improved the diagnostic yield when compared to bronchoscopy and transbronchial biopsy [26]. Several studies have shown that delays occur and if the first tissue procedure fails [27–29], complications and costs increases [30], suggesting that procedure for collection of tissue samples should be discussed at a multidisciplinary tumor board [31].

There are several limitations to our study. It is a single-center study and the mechanisms for delay may not necessarily be relevant for other hospitals or other health care systems. E.g. the health care system in Norway is public, and the PET CT availability may have been more limited during the study period than in otherwise comparable health care systems. But even if the reasons for delay may vary between hospitals, we believe that our study presents an approach for analyzing delays that might be useful also in other organizations. Furthermore, we decided to limit our study to stage I-II patients due to the complexity and heterogeneity of patients with more advanced stage and did not include data on patients who underwent similar diagnostic work-up but were not diagnosed with lung cancer. We have not included analyses of time trends due to the significant impact of availability of PET CT. When the scanner was installed at our hospitals, the waiting time for PET CT reports was shortened, but more patients underwent the procedure. Furthermore, there were no organizational changes during the study report that would impact the treatment time.

We are not aware of any internationally accepted definition of an optimal pathway for diagnostic workup for lung cancer. Our definition is only based on what we consider most time-effective and may be questioned. There were quite many bronchoscopies that did not add any information or resulted in a definitive diagnosis of cancer. However, unsuspected endobronchial involvement occurs, and some recommend that a bronchoscopy should be one of the first diagnostic procedures when lung cancer is suspected [32, 33]. Furthermore, it is not always obvious which method for tissue sampling that has a highest chance of success, and bronchoscopy entails less risk of complications than a CT guided transthoracic biopsy. We do, however, argue that all relevant imaging including a PET CT scan should be performed before any invasive procedure including bronchoscopy to better plan the procedure and increase the chances for a positive yield from the tissue sampling.

Norwegian guidelines are not explicit on whether PET CT should be done before or after a diagnosis has been confirmed [9]. Access to PET CT is limited, and many patients have to travel long distances for a PET CT. But if reducing time to treatment is the highest priority, our data strongly indicate that a PET CT should be performed as soon as possible after the CT scan in preliminary stage I-II.

Referring patients for treatment before all procedures have been completed may be more controversial. In our cohort, the treatment plan changed in only 2% of cases after PET CT, but will still cause cancellations of planned treatment, which requires good administrative systems to fully utilize the capacity in operating theatres and radiotherapy departments. Finally, the impact on timelines of applying our optimal pathway is simulated and not validated in an intervention trial.

The main strength of our study is that we have performed a comprehensive review of the individual patients’ trajectories from the individual medical records. Most other studies investigating timeliness of lung cancer care, utilize registry data and investigate associations between demographic data, hospital and patient characteristics with timelines [10, 34–41]. Our study group is multidisciplinary (pulmonologists, an oncologist and a gynecologist not involved in diagnosis or treatment of lung cancer), and there were few changes in the staff of pulmonologists who worked at our hospital during the study period.

We believe that our study strongly suggests that the time until start of treatment can be greatly reduced by analyzing current pathways for diagnostic workup and applying a more rational pathway without adding more resources. E.g. the time until the first appointment has later been reduced through better organization. Furthermore, the number of diagnostic procedures would have been reduced if the optimal pathway had been applied, which would reduce the burden on patients and consequently the risk of severe complications. This might be of even greater benefit of patients with delays due to medical reasons (i.e. significant comorbidity) who were excluded from the present study even if they still would not be able to start treatment within 42 days. Still, time until treatment would have been further reduced if waiting time for tissue sampling procedures, radiology- and pathology reports, surgery and radiotherapy were shortened, indicating that at some point, more resources are needed in order to further reduce the time until treatment.

The most common role of tumor board meetings seems to be to discuss treatment alternatives, and thus facilitate a fast and correct treatment [22, 42]. Our study indicate that the patients can in most cases be referred for treatment while the tissue procedures are being performed since the treatment recommendation will change in very few. The tumor board might also play a role in selecting the most correct diagnostic procedures, suggesting that patients should be discussed by a tumor board after the initial imaging and physical examinations have been performed. It is possible, though, that applying our pathway including the early discussion at a tumor board and early referral for treatment is only applicable at larger hospitals. More frequent meetings of the tumor board might reduce the time to treatment even more, but in our cohort, the effect of better planning would have a much greater impact on the timelines.

It remains unclear whether shortening the time until treatment has a positive impact on the treatment outcomes, and one might argue that since the median time to treatment was 46.5 days, there is no benefit of reaching the goal of 42 days. However, the goal is that 70% of patients should start treatment within 42 days. Thus, our hospital was far behind this goal even for the least complicated patients analyzed in the present study.

## Conclusions

Only 40% of preliminary stage I-II lung cancer patients started treatment within the recommended 42 days at our hospital. When applying the current waiting time for PET CT (≤7 days), the proportion increased to 48%. If also a more optimal pathway had been applied, the proportion could increase to 80% and the number of diagnostic procedures could be reduced with 16%.

## Data Availability

The datasets used and analysed during the current study are available from the corresponding author on reasonable request.

## Abbreviations

CT: Computer tomography
EBUS TBNA: Endobronchial ultrasound transbronchial needle aspiration
PET CT: Positron emission tomography computer tomography
TTNB: Transthoracic needle biopsy
